# A potential role for Dkk-1 in the pathogenesis of osteosarcoma predicts novel diagnostic and treatment strategies

**DOI:** 10.1038/sj.bjc.6604069

**Published:** 2007-11-06

**Authors:** N Lee, A J Smolarz, S Olson, O David, J Reiser, R Kutner, N C Daw, D J Prockop, E M Horwitz, C A Gregory

**Affiliations:** 1 Department of Medicine, Center for Gene Therapy, Tulane University Health Sciences Center, 1430 Tulane Avenue, New Orleans, LA 70112, USA; 2 University of Illinois College of Medicine, Chicago, IL 6061, USA; 3 Department of Pathology, Tulane Hospital and Clinic, Tulane Avenue, New Orleans, LA 70112, USA; 4 Vector Core, Louisiana State University Health Sciences Center, 1901 Perdido Street, Suite 322, New Orleans, LA 70112, USA; 5 Department of Oncology, St Jude Children's Research Hospital, 332 N Lauderdale, Memphis, TN 38105-2794, USA; 6 Children's Hospital of Philadelphia, Abramson Research Center, 1116D, 3615 Civic Center Boulevard, Philadelphia, PA 19104, USA; 7 Division of Bone Marrow Transplantation, St Jude Children's Research Hospital, 332 N Lauderdale, Memphis, TN 38105-2794, USA

**Keywords:** mesenchymal stem cell, osteosarcoma, Dkk-1, MSC

## Abstract

Canonical Wnt signalling is an osteoinductive signal that promotes bone repair through acceleration of osteogenic differentiation by progenitors. Dkk-1 is a secreted inhibitor of canonical Wnt signalling and thus inhibits osteogenesis. To examine a potential osteoinhibitory role of Dkk-1 in osteosarcoma (OS), we measured serum Dkk-1 in paediatric patients with OS (median age, 13.4 years) and found it to be significantly elevated. We also found that Dkk-1 was maximally expressed by the OS cells at the tumour periphery and *in vitro*, Dkk-1 and RANKL are coexpressed by rapidly proliferating OS cells. Both Dkk-1 and conditioned media from OS cells reduce osteogenesis by human mesenchymal cells and by immunodepletion of Dkk-1, or by adding a GSK3*β* inhibitor, the effects of Dkk-1 were attenuated. In mice, we found that the expression of Dkk-1 from implanted tumours was similar to the human tumour biopsies in that human Dkk-1 was present in the serum of recipient animals. These data demonstrate that systemic levels of Dkk-1 are elevated in OS. Furthermore, the expression of Dkk-1 by the OS cells at the periphery of the tumour probably contributes to its expansion by inhibiting repair of the surrounding bone. These data demonstrate that Dkk-1 may serve as a prognostic or diagnostic marker for evaluation of OS and furthermore, immunodepletion of Dkk-1 or administration of GSK3*β* inhibitors could represent an adjunct therapy for this disease.

Mesenchymal stem cells (MSCs) from the bone marrow are progenitors of osteoblasts, the cells that build and maintain bone tissue. The process of osteoblast differentiation, and the subsequent repair of bone is driven by canonical Wingless (Wnt) signalling within the MSC (Cadigan and Nusse, 1997; Kikuchi, 2000; Huelsken and Birchmeier, 2001; Pandur *et al*, 2002; Bain *et al*, 2003; Rawadi *et al*, 2003; Bodine *et al*, 2004; Bennett *et al*, 2005; Gregory *et al*, 2005a, 2005b; Holmen *et al*, 2005; Hartmann, 2006; Krishnan *et al*, 2006).

In the canonical Wnt signalling pathway, Wnt ligands bind to the transmembrane receptor frizzled (Frz) and the co-receptor lipoprotein related proteins 5 and 6 (LRP-5/6) on the surface of the target cell. Activation of Frz recruits the cytoplasmic-bridging molecule, disheveled, so as to inhibit the action of glycogen synthetase kinase-3*β* (GSK3*β*). Inhibition of GSK3*β* decreases phosphorylation of *β*-catenin, preventing its degradation by the ubiquitin-mediated pathway. The stabilised *β*-catenin acts on the nucleus by activating TCF/LEF-mediated transcription of target genes that elicit a variety of effects including induction of differentiation and proliferation (Cadigan and Nusse, 1997; Kikuchi, 2000; Huelsken and Birchmeier, 2001; Pandur *et al*, 2002).

The presence of Wnt signalling inhibitors such as Dkk-1 (Glinka *et al*, 1998; Krupnik *et al*, 1999; Nusse, 2001; Mao *et al*, 2002; Kawano and Kypta, 2003; Li *et al*, 2006; Morvan *et al*, 2006) can disrupt the repair of bone and its secretion by tumours into the bone can lead to irreparable damage to the tissue. Examples include multiple myeloma (Tian *et al*, 2003) and some forms of prostate cancer (Hall *et al*, 2005). Damage of the bone by malignancy can increase the severity of the disease by providing a permissive microenvironment for tumour growth and metastatic events. In multiple myeloma, Dkk-1 is readily detectable in the blood of individuals in the later stages of the disease who have characteristic osteolytic bone lesions (Tian *et al*, 2003).

Given that high levels of Dkk-1 are also expressed by rapidly dividing osteosarcoma (OS) cells *in vitro* (Gregory *et al*, 2003, 2005a, 2005b), we measured the level of Dkk-1 in the serum of paediatric patients with OS (median age, 13.4 years) and found it to be significantly elevated when compared with healthy controls. The aim of this study therefore, was to investigate the significance of such a finding in OS. Dkk-1 and Receptor Activator for Nuclear Factor-*κ*B Ligand (RANKL) were found to be maximally expressed by rapidly dividing OS cells *in vitro* and by cells at the periphery of the solid tumour *in vivo*. Since Dkk-1 and RANKL inhibit osteogenic differentiation and activate osteoclasts, respectively, these proteins probably contribute to tumour expansion by inhibiting repair of the surrounding bone while simultaneously accelerating resorption. Employing *in vitro* assays, we examined the possibility that immunodepletion of Dkk-1 or administration of GSK3*β* inhibitors could represent an adjunct therapy for this disease by improving osteogenic tissue repair adjacent to the tumour.

## MATERIALS AND METHODS

### Human biomaterial acquisition

The handling and acquisition of human-derived biomaterials were performed in accordance with the Institutional Review Boards and Ethics Committees of Tulane University Hospital and Clinic (New Orleans, LA, USA) and St Jude Children's Hospital (Memphis, TN, USA). The OS serum samples were acquired from the tissue bank of St Jude Children's Hospital, and the control group samples were collected from unaffected individuals at Tulane University Hospital and Clinic. Human MSCs were acquired from the Tulane Adult MSC Distribution Core (Tulane University, New Orleans, LA, USA) and cultured in accordance with their protocols.

### ELISA assays

Frozen serum samples from newly diagnosed patients with OS were acquired from St Jude Children's Hospital under the supervision of Dr N Daw and Dr E Horwitz. Serum samples from unaffected individuals were drawn and prepared at Tulane University Hospital and Clinic. Enzyme-linked immunosorbent assays (ELISAs) were performed using a polyclonal duo set (R&D Systems, Minneapolis, MN, USA, catalogue no. AF1096) consisting of a goat anti-human Dkk-1 antibody and a biotinylated sample of the same serum. Microtitre plates (Nunc Immunosorp, Rochester, NY, USA) were coated with 100 *μ*l of a 1 *μ*g ml^−1^ solution of the capture antibody for 15 h at 4°C, then blocked for 2 h at 21–25°C in PBST (phosphate-buffered saline containing 0.1% (v/v) Tween 20) containing 4% (v/v) bovine serum albumin. Samples were diluted initially at 1 : 3 then up to 1 : 10 in PBST depending on the levels of Dkk-1 in the sample. Plates were washed three times prior to loading onto the plate in 100 *μ*l aliquots. Samples were incubated for 15 h at 4°C in the plates, wells were then washed three times in PBST. One hundred microlitres of a 0.5-*μ*g ml^−1^ detection antibody was then added to each well followed by incubation for 2 h at 21–25°C. The wells were then washed three times in PBST, followed by addition of a 1 : 2000 dilution of streptavidin-conjugated horseradish peroxidase (Pierce, Rockford, IL, USA). After a final wash step, the wells were developed by addition of 2,2′-azino-bis(3-ethylbenzthiazoline-6-sulphonic acid) or tetramethlybenzidine. After stopping the reactions with 5 M NaOH or 2 M H_2_SO_4,_ respectively (Sigma, Poole, Dorset, UK), wells were read at 405 or 450 nm by automated plate reader (Fluostar; BMG Labtech, Durham, NC, USA). The linear range of the assay was 0.2–20 ng ml^−1^ and maximum variation between assays was 5% of the mean.

### Histology and immunocytochemistry

Osteosarcoma tumours, synthetic tumour constructs or monolayer cells were fixed in formalin and processed as paraffin blocks if necessary. For histology, 8-*μ*m sections were prepared, deparaffinised and rehydrated, then stained with haematoxylin–eosin (Sigma). For immunohistochemistry, after an acidic antigen retrieval step (R&D Systems), sections were blocked and incubated in the presence of a 1 : 800 dilution of goat anti-human Dkk-1 or monoclonal anti-RANKL antibody (R&D Systems). Monolayer cultures were directly subjected to immunocytochemistry after fixation. Alexafluor 594 or 488 conjugated secondary (Invitrogen, Carlsbad, CA, USA) antibodies were employed to detect antigen binding.

### Alkaline phosphatase assays

Alkaline phosphatase assays were performed on monolayers in six-well format as previously described (Gunn *et al*, 2005). Briefly, MSCs were seeded at a density of 5000 cells per cm^2^ and cultured for 10 days in osteoinductive media. Osteoinductive media consisted of alpha MEM containing 20% (v/v) fetal bovine serum (Atlanta Biologicals, Norcross, GA, USA), 100 unit ml^−1^ penicillin, 1 *μ*g ml^−1^ streptomycin, 4 mM L-glutamine, 5 mM *β*-glycerophosphate (Sigma), 100 *μ*g ml^−1^ ascorbate-2-phosphate (Sigma) and the appropriate concentration of Dkk-1 or bromo-indirubin-3′-mono-oxime (BIO; Calbiochem, La Jolla, CA, USA) suspended in dimethylsulphoxide (Sigma). Unless otherwise stated, all cell culture reagents were purchased from Invitrogen. Recombinant human Dkk-1 preparation was carried out as previously described from a stably expressing COS cell line (Gunn *et al*, 2005).

### Mineralisation assays

All reagents were purchased from Sigma. In the six-well format, MSCs were cultured at high density (500 cells per cm^2^) in the presence of osteoinductive media, but in the absence of steroids, for 15 days with or without 500 ng ml^−1^ recombinant Dkk-1. Thereafter, the media was transferred to osteoinductive media with 10^−8^ M dexamethasone for a further 7 days. The monolayers were then washed in PBS, fixed for 10 min in phosphate-buffered formalin, then stained with the calcium-binding dye, Alizarin Red S. Monolayers were photographed using a Nikon Eclipse TE200 inverted microscope fitted with a Nikon DXM1200F digital camera.

### Cell counting assays

Cells were counted based on nucleic intercalation of a fluorescent dye (CyQuant, Invitrogen). Assays were performed as previously described (Gregory *et al*, 2003, 2005b).

### Western blotting

Western blots were performed on Triton X-100 (Sigma) insoluble extracts of cells using the goat anti-human Dkk-1 or monoclonal anti RANKL antibody (R&D Systems). Controls for actin and glyceraldehyde 3-phosphate dehydrogenase (GAPDH) were employed as previously described (Gregory *et al*, 2005b).

### Immunoaffinity depletion

The inhibitor Dkk-1 was depleted from the medium by antibody incubation and protein A/G-mediated depletion as previously described (Gregory *et al*, 2003). Immunodepletion was carried out using the rabbit anti-Dkk-1 polyclonal antiserum described in Gregory *et al* (2003) or the goat anti-Dkk-1 polyclonal acquired from R&D Systems. Protein A (for rabbit) and protein G (for goat) were conjugated to sepharose beads (Amersham Pharmacia Biotech, Piscataway, NJ, USA).

### Cell labelling

The lentiviral construct encoding the dsRed fluorescent protein coupled to the mitochondrial localisation sequence of human cytochrome *c* oxidase subunit VIII was prepared using standard protocols by virus core facility at Louisiana State University viral vector core (Marino *et al*, 2003; Zhang *et al*, 2004). Proliferating MG63 cells were exposed to the virus at a multiplicity of infection of 80 in the presence of 9 *μ*g ml^−1^ polybrene for 18 h. After 4 days, approximately 50% of the cells expressed the fluorescent protein. Expressing cells were selected by fluorescent-activated cell sorting (Facsvantage SE; Becton Dickinson, Franklin Lakes, NJ, USA).

### Constructs

Labelled cells were suspended in 1 ml of a 2 × reconstitution of dried human plasma (Sigma) and mixed with an equal volume of thromboplastin C (Plastinex; Fisher Life Sciences, Pittsburg, PA, USA). The mixture was transferred to a 10 mm × 20 mm chamber slide for gelling. Clotting was allowed to proceed for 2–4 h, and then the appropriate experimental medium preparation was added to cover the solid construct until implantation.

### Implantation

Experiments were conducted in accordance with Tulane University Animal Use and Care Committee regulations. Fibrin constructs were implanted subcutaneously between the scapulae of anaesthetised nude mice. A 10-mm incision was made longitudinally between the scapulae, and a small cavity was made between the dermis of the skin and the fascia below to accommodate the constructs, which were 10 mm^2^. The incision was then closed by 2–3 sutures, and sealed (Vetbond; 3M, St Paul, MN, USA). After 5 days, the sutures were removed. Seven days thereafter, the animals were placed under anaesthesia, euthanised by cardiac exsanguination, and serum was prepared from the blood. The implants were removed for genomic DNA extraction. Genomic DNA was extracted from the tissue by phenol chloroform extraction (Trizol, Invitrogen) and subjected to quantitative real-time PCR for the dsRed gene using the following primers: forward, ACTACAAGAAGCTGTCCTTCC and reverse, TTCACGCCGATGAACTTCACC. Reactions were cycled on an ABI PRISM 7700 Sequence Detector (Applied Biosystems) for 40 cycles with the annealing temperature set to 60°C. Products were detected by fluorescence intercalation (SYBR Green, Applied Biosystems) and validated by gel electrophoresis and melting curve analysis.

## RESULTS

**Figure 1:**
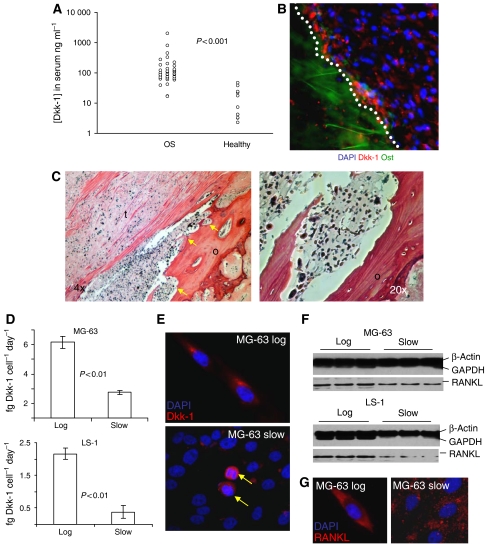
(**A**) Scatter plot of the circulating Dkk-1 levels in OS patients and unaffected individuals. Measurements were performed by ELISA. The difference between Dkk-1 levels in patients *vs* healthy controls was significant (*P*<0.00002) when compared using a Mann–Whitney *U*-test. (**B**) A sectioned OS immunostained for the detection of Dkk-1 (red). Dkk-1 expression is maximal at the border zone between the osteoid tissue (green autofluorescence) and the tumour cells (white dotted line). Nuclei are stained with 4′-6-diamidino-2-phenylindole (DAPI; blue). (**C**) The OS is sectioned and stained with haematoxylin–eosin. The osteoid (o) is infiltrated with tumour cells (t), resulting in rough zones of osteoid remodelling (arrowed). A high-power micrograph of a zone of osteoid degradation is provided on the right. (**D**) Secretion of Dkk-1 by rapidly dividing, low-density cultures (log) and slowly dividing confluent cultures (slow) of MG63 and LS1 OS cells. Measurements were made by ELISA, values represent the mean (*n*=6), and error bars represent s.d. *P*-values were calculated by two-tailed Student's *t*-test. (**E**) Monolayers of MG63 cells at high (slow) and low (log) density immunostained for Dkk-1. Note that staining is maximal in the low-density cultures and in the high-density cultures, Dkk-1 staining is confined to those cells undergoing mitosis (arrowed). The DNA is stained with DAPI (blue). (**F**) Western blot of membrane isolates derived from MG63 and LS1 OS cells. The control lanes (upper) were simultaneously incubated with an anti-*β*-actin and anti-GAPDH antibodies since enrichment for insoluble, membrane bound, *β*-actin confirms that the membranes have been efficiently recovered at the expense of cytosolic components such as GAPDH. Membrane-bound RANKL was detected on the same stripped blot (lower) with an anti-RANKL antibody. (**G**) Monolayers of nonpermeabilised MG63 cells at high (slow) and low (log) density immunostained for RANKL. Note that staining is maximal in the low-density cultures and punctuates in the high-density cultures. The DNA is stained with DAPI (blue).

We measured the levels of Dkk-1 in the serum of newly diagnosed individuals with OS by ELISA and found that the mean levels were elevated (*P*<0.00002, Mann–Whitney *U*-test, two tails) in affected individuals (range: 16.84–2210.14 ng ml^−1^, mean 191.91 ng ml^−1^, median 90.53 ng ml^−1^) when compared to unaffected individuals (range: 2.28–43.38 ng ml^−1^, mean 21.66 ng ml^−1^, median 19.67 ng ml^−1^). Although the control group (*n*=12) was smaller than the OS group (*n*=37), and the median age of the unaffected individuals was slightly higher (by approximately 7 years), the control Dkk-1 values were similar to the normal levels reported by Tian *et al* (2003), who demonstrated that elevated levels of serum Dkk-1 were coincident with the osteolytic lesions seen in most cases of multiple myeloma (Figure 1A). The Dkk-1 levels in the affected individuals were somewhat higher than those documented in the study by Tian *et al* (2003) with the highest levels in the micromolar range. Immunohistochemical staining of excised tumour biopsies demonstrated that Dkk-1 was expressed maximally at the periphery of the tumour, adjacent to the hosts' bone tissue (Figure 1B). Upon histological examination of serial sections of excised tumour tissue, the areas that stained most intensely for Dkk-1 were accompanied by extensive remodelling. The border of the adjacent osteoid was irregular, with frequent penetration of many tumour cells, consistent with a destructive OS (Figure 1C).

The expression of Dkk-1 by two OS cell lines; MG63, a well-characterised osteogenic sarcoma and LS1, a cell line derived from an excised OS, was examined in more detail in tissue culture experiments. Dkk-1 was found to be maximally secreted by cells rapidly proliferating in sparsely populated monolayers but was significantly reduced as proliferation slowed and the monolayer became more confluent (Figures 1D and E). Interestingly, Dkk-1 expression in the higher density monolayers was confined to a small fraction of cells that were clearly in the metaphase of cell division (Figure 1E). Also, the potent upregulator of osteoclast activity, RANKL (Blair *et al*, 2006) mirrored the expression of Dkk-1 in OS cells, but it was exclusively detected as the membrane bound form rather than the secreted form (Figures 1F and G). On the basis of these observations, we hypothesised that the expression of Dkk-1 and RANKL at the periphery of the tumour was necessary for osteogenic remodelling as the tumour expands. The presence of high levels of Dkk-1 and RANKL facilitate expansion by allowing the proliferative cells at the periphery of the tumour to accelerate bone resorption through expression of RANKL while inhibiting osteoid repair through the action of Dkk-1.

**Figure 2:**
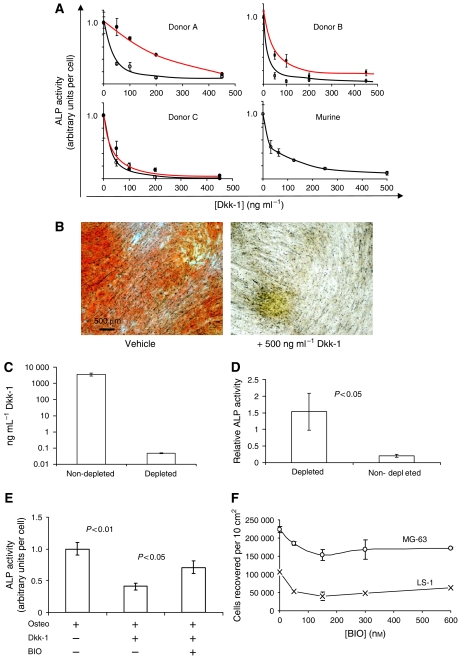
(**A**) Osteogenic differentiation of MSCs in the presence of Dkk-1. Results from cells derived from three human donors and pooled murine donors are presented. Osteogenic differentiation is presented as a function of membrane ALP activity, an early marker of osteogenesis. Measurements are normalised to control levels of activity, designated 1.0. The black lines represent MSCs prepared from the fluid component of bone marrow, and the red lines represent MSCs prepared from bone spicules filtered from the aspirates. Dkk-1 exposure causes a dose-dependent inhibition of alkaline phosphatase activity. (**B**) Alizarin Red stained, long-term cultures of osteogenic MSCs in the presence and absence of Dkk-1. Calcium detection by Alizarin Red S demonstrates that Dkk-1 inhibits mineralisation of the cultures. (**C**) Immunodepletion of Dkk-1 from MG63 OS conditioned medium through incubation with a polyclonal antibody against Dkk-1. The Dkk-1–antibody complexes were removed from the medium by protein A affinity chromatography, then the medium was assayed by ELISA. (**D**) Osteogenic differentiation by MSCs in the presence of nondepleted and Dkk-1 immunodepleted conditioned medium from MG63 OS cells. Representative results from one out of three donors are presented. Measurements were achieved by ALP assay, values represent the mean (*n*=6), and error bars represent s.d. *P*-values were calculated by two-tailed Student's *t*-test. (**E**) Osteogenic differentiation by MSCs in the presence of Dkk-1 and with or without the GSK3*β* inhibitor BIO. (**F**) The effect of a range of BIO doses on the proliferation of OS cells. Cell numbers were evaluated by fluorescent nucleic acid intercalation assay.

The putative osteoinhibitory effect of recombinant Dkk-1 was tested in a tissue culture model of osteogenic differentiation by human primary MSCs. At concentrations equivalent to those measured in OS patients, Dkk-1 inhibited the expression of the osteogenic marker, alkaline phosphatase, by MSCs in a dose-dependent manner when added to osteogenic cultures (Figure 2A). The effect was observed in MSCs from three donors and pooled murine MSCs. In two donors (Figure 2A), MSCs cultured directly from bone spicules (red) were more resistant to Dkk-1 than those cultured from the fluid component of the marrow (black), suggesting that the MSCs were probably osteogenically preconditioned by the niche of the bone tissue. The observation that serum alkaline phosphatase is occasionally upregulated in OS patients, suggests that Dkk-1 acts to prevent differentiation of progenitor cells, but does not affect the release of alkaline phosphatase from preexisting osteoblasts at sites of bone remodelling. To examine the effect of Dkk-1 on late-stage osteogenesis by MSCs, the cells were cultured in with *β*-glycerophosphate and ascorbic acid for 15 days, and then in the presence of dexamethasone for a further 7 days to induce calcification. The monolayers were then fixed and stained with the calcium-binding dye, Alizarin Red S. The presence of 500 ng ml^−1^ Dkk-1 in the 15-day preincubation step inhibited calcification of the monolayers when compared with controls (Figure 2B).

When media were conditioned by MG-63 OS cells and added to osteogenic cultures of MSCs, osteogenic inhibition occurred (Figure 2D), and this effect was attenuated upon immunodepletion of Dkk-1 from the medium (Figures 2C and D). Dkk-1 inhibits the Wnt pathway by sequestering the Wnt co-receptor, LRP6 and preventing the Wnt-induced coalescence of Frz and LRP6 at the membrane. The downstream effect of the LRP6/Wnt/Frz complex is to inhibit GSK3*β*, reduce phosphorylation of *β*-catenin and prevent its degradation by the proteosomal machinery. Stabilised *β*-catenin complexes with TCF/LEF mediates transcription of target genes, in this case, osteogenic genes. The presence of a pharmaceutical inhibitor of GSK3*β* would be predicted to elicit the same effect as Wnt signalling, irrespective of the level of Dkk-1 in the system. Osteogenic cultures were therefore prepared in the presence of Dkk-1 with or without the GSK3*β* inhibitor, BIO. The presence of BIO reduced the osteoinhibitory effect of Dkk-1 (Figure 2E). Since Wnt signalling has been implicated in the induction of oncogenesis, we tested the effect of escalating doses of BIO on MG-63 and LS-1 cell proliferation. At the concentrations tested, there was no significant induction of proliferation by BIO (Figure 2F).

**Figure 3:**
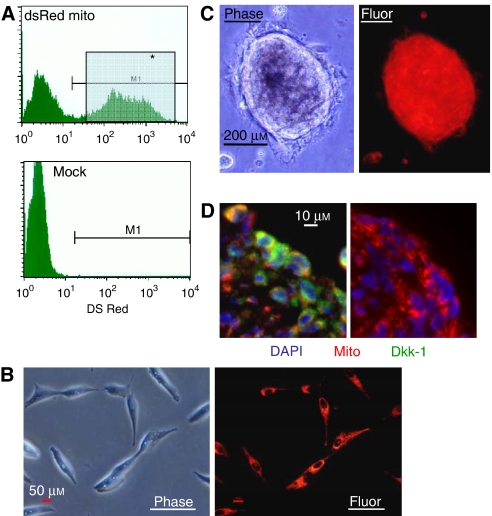
(**A**) Fluorescence-activated cell sorting of transduced cells expressing mitochondrially localised red fluorescent protein. The cells from the gate designated M1 were used in subsequent experiments. (**B**) Micrographs of the labelled MG63 OS cells. (**C**) Micrographs of the tumour spheres derived from culture in clotted human plasma. (**D**) A sectioned tumour sphere (red) immunostained for the detection of Dkk-1 (green). Nuclei are stained with DAPI (blue). The isotype control is presented on the right.

**Figure 4:**
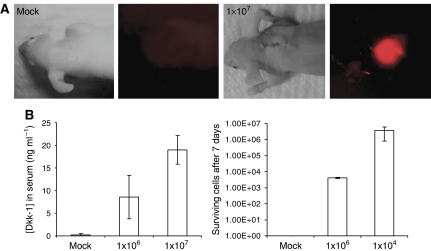
(**A**) Live animal fluorescence imaging of an implanted construct containing labelled MG63 OS cells. (**B**) Evaluation of human Dkk-1 levels in the blood of implanted animals after 1 week. The *x*-axis represents the initial number of implanted cells. Measurements were achieved by ELISA on mouse serum, values represent the mean (*n*=4, two men and two women), and error bars represent s.d. *P*-values were calculated by two-tailed Student's *t*-test.

We established an OS model to recapitulate some of the effects of Dkk-1 and OS *in vivo*. MG-63 cells were labelled by lentiviral transduction of a fusion gene combining the mitochondrial localisation sequence of cytochrome *c* oxidase with the fluorescent protein, dsRed (Figures 3A and B). Upon suspension culture in the presence of clotted human plasma, after 24–48 h, the cells formed tumour spheroids that ranged from approximately 10 to 5000 cells in diameter within the fibrin gel (Figure 3C). Smaller spheroids expressed Dkk-1 throughout, but the larger structures adopted an expression pattern for Dkk-1 that mimicked the tumour biopsies (Figure 3D) with the maximal level of expression at the periphery. To examine Dkk-1 expression by OS cells *in vivo*, fibrin constructs containing 1 million and 10 million labelled MG63 cells were implanted in nude mice against the upper thoracic vertebrae. After 1 week, the constructs were clearly visible by live animal fluorescent imaging (Figure 4A). Furthermore, human Dkk-1 could be detected in the blood of implanted animals when assayed by ELISA (Figure 4B) and the level of circulating Dkk-1 correlated with the number of surviving cells in the construct. After 2 and 4 weeks post implantation, the number of MG63 cells present in the recipients had reduced resulting in a concomitant reduction of systemic Dkk-1 (data not shown). The reason for the reduced viability of the cells over extended implantation periods is unclear, but macrophage mediated destruction of implants in immunocompromised mice has been reported in the literature (Xia *et al*, 2004). In spite of the hosts' response to the implanted cells, human Dkk-1 could be detected in the blood of the recipient mice, demonstrating that tumour-derived Dkk-1 escapes into the blood stream. It is unclear at this point whether the elevated systemic Dkk-1 in OS patients is derived solely from the tumour, since the human Dkk-1 circulating in the blood of recipient mice was much lower than the mean levels detected in the blood of the human OS patients. It is possible, however, that the host tissue interacts with the tumour, resulting in upregulation of the expression of Dkk-1, a phenomenon observed in the case of multiple myeloma (Gunn *et al*, 2005; Corre *et al*, 2007). The host microenvironment in the patients may be more readily affected by the tumour than the surrounding mouse tissue accounting for the reduced levels of Dkk-1 in the mouse blood when compared to the human blood. At any rate, the presence of OS cells in both humans and the recipient mice resulted in elevated circulating human Dkk-1, suggesting that the molecule could represent a valuable diagnostic tool. The correlation of tumour load with Dkk-1 levels also suggests that the assay also has potential for measuring the relative size and severity of such tumours.

## DISCUSSION

These data strongly suggest that the canonical Wnt inhibitor Dkk-1 is highly expressed by OS tumours at levels that become systemically detectable in humans. Furthermore, the *in vivo* data demonstrate that the level of Dkk-1 detectable in blood is proportional to the number of surviving OS cells in the tumour. Assays of Dkk-1 secretion could therefore represent a useful diagnostic and prognostic tool for the evaluation of OS patients. Dkk-1 is also upregulated by other malignant cell lines (Wirths *et al*, 2003; Forget *et al*, 2007), suggesting that serum Dkk-1 measurements may be useful for the evaluation of other types of malignancy, but the significance of Dkk-1 expression is not presently clear in tumours that do not affect bone. Although the data presented here demonstrate that Dkk-1 may contribute to OS pathogenesis by preventing repair of the surrounding osteoid as the tumour expands, Dkk-1 may act in an autocrine manner on the tumour cells too. Noteworthy are the observations that exposure of MG63 and LS1 OS cells to high concentrations of BIO reduces proliferation (Figure 2F), and immunosequestration of Dkk-1 transiently slowed the proliferation of MG63 cells *in vitro* (Gregory *et al*, 2003). It is unlikely that Dkk-1 can solely act as a mitogen, but its presence at a critical threshold may serve as a checkpoint that permits proliferation of OS cells. One attractive hypothesis is that Dkk-1 has to be present to prevent inappropriate differentiation during a rapid burst of mitosis and a collateral effect of this is to prevent osteoid repair by progenitor cells.

In instances of osteolytic tumours, the presence of Dkk-1 would be predicted to exacerbate lesion formation through inhibition of Wnt-dependent osteogenesis. This certainly seems probable since a recent study has demonstrated that prostate tumours-expressing high levels of Dkk-1 produce more extensive local bone destruction compared to controls that express lower levels (Hall *et al*, 2005). Reducing the osteoinhibitory effects of Dkk-1 would therefore be predicted to reduce local bone damage, and as a result, probably reduce the expansion of the tumour. This could be achieved by pharmaceutical inhibition of GSK3*β*, by inhibition of the proteasomal degradation pathway, or by antibody-mediated sequestration of Dkk-1. The osteoinductive properties of GSK3*β* inhibitors have been demonstrated both *in vivo* and *in vitro* by numerous investigators (Gregory *et al*, 2006), proteasomal inhibitors have been shown to reduce Dkk-1 and RANKL levels (Terpos *et al*, 2006), and the benefits of administration of an anti-Dkk-1 antibody have recently been demonstrated in a murine model of multiple myeloma (Yaccoby *et al*, 2007). However, it remains to be seen whether the induction of Wnt signalling either by GSK3*β* inhibition, or by antibody administration may affect the metastatic potential of OS cells since Wnt signalling and/or *β*-catenin upregulation has been shown to be a key regulator of migration in prostate tumours, multiple myeloma cells and also in OS cells (Iwaya *et al*, 2003; Qiang *et al*, 2005; Hall *et al*, 2006). Although we did not detect signs of pulmonary metastasis in this study, further investigation employing highly metastatic OS cell lines should be performed in future work.

Irrespective of concerns regarding the use and safety of GSK3*β* inhibitors, proteasome inhibitors or antibodies directed towards Dkk-1 as an adjunct treatment for OS, the diagnostic utility of serum Dkk-1 is strongly supported by this study. Since tumour-derived Dkk-1 was present in the blood of mice at levels proportional to the number of surviving tumour cells, Dkk-1 may serve as a powerful and noninvasive tool for the evaluation of patients with OS.
